# 4-O-Methylascochlorin Synergistically Enhances 5-Fluorouracil-Induced Apoptosis by Inhibiting the Wnt/β-Catenin Signaling Pathway in Colorectal Cancer Cells

**DOI:** 10.3390/ijms25115746

**Published:** 2024-05-25

**Authors:** Min-Young Jo, Yun-Jeong Jeong, Kwon-Ho Song, Yung Hyun Choi, Taeg Kyu Kwon, Young-Chae Chang

**Affiliations:** 1 Research Institute of Biomedical Engineering and Department of Cell Biology, Daegu Catholic University School of Medicine, Daegu 42472, Republic of Korea; 2 Department of Biochemistry, College of Korean Medicine, Dong-Eui University, Busan 47227, Republic of Korea; 3 Department of Immunology, School of Medicine, Keimyung University, Daegu 42601, Republic of Korea

**Keywords:** 4-O-methlyascochlorin, 5-fluorouracil, synergistic effects, drug-resistance, β-catenin

## Abstract

4-O-Methyl-ascochlorin (MAC), a derivative of the prenyl–phenol antibiotic ascochlorin extracted from the fungus *Ascochyta viciae*, shows anticarcinogenic effects on various cancer cells. 5-Fluorouracil (5-FU) is used to treat colorectal cancer (CRC); however, its efficacy must be enhanced. In this study, we investigated the molecular mechanisms by which MAC acts synergistically with 5-FU to inhibit cell proliferation and induce apoptosis in CRC cells. MAC enhanced the cytotoxic effects of 5-FU by suppressing the Akt/mTOR/p70S6K and Wnt/β-catenin signaling pathways. It also reduced the viability of 5-FU-resistant (5-FU-R) cells. Furthermore, expression of anti-apoptosis-related proteins and cancer stem-like cell (CSC) markers by 5-FU-R cells decreased in response to MAC. Similar to MAC, the knockdown of CTNNB1 induced apoptosis and reduced expression of mRNA encoding CRC markers in 5-FU-R cells. In summary, these results suggest that MAC and other β-catenin modulators may be useful in overcoming the 5-FU resistance of CRC cells.

## 1. Introduction

Colorectal cancer (CRC) is one of the most common cancers worldwide [1]. Despite recent advances in treatment, the prognosis of CRC patients remains poor due to high rates of recurrence and metastasis [2,3]. Currently, the most widely used chemotherapeutic molecule for CRC patients is 5-fluorouracil (5-FU) [4]. 5-FU-based agents such as FOLFOX and FOLFIRI show beneficial effects against advanced CRC, but drug resistance can lead to poor prognosis [5]. Accumulating evidence suggests that cancer cells that have become resistant to 5-FU exhibit stemness [6], leading to enhanced anti-apoptotic, and metastatic characteristics [7,8]. Hence, strategies are urgently needed to improve the clinical outcomes of 5-FU therapy and better understand the mechanisms underlying 5-FU resistance.

Activation of Wnt/β-catenin signaling leads to CRC via hyperproliferation and oncogenic transformation of intestinal epithelial cells [9]. This pathway is abnormally active in CRC. Over 80% of CRC patients have mutations in the Wnt antagonist Adenomatous Polyposis Coli (APC) [10,11,12]. Activation of Akt/mTOR signaling, which is implicated in the activation of β-catenin, also promotes differentiation, protein synthesis, proliferation, and survival of CRC cells [13,14,15]. In addition, the Wnt/β-catenin and Akt/mTOR signaling pathways support the survival of cancer stem-like cells (CSCs), which may contribute to drug resistance [16].

Recent studies have emphasized the critical role of CSCs, which possess continuous self-renewal capabilities, in tumor initiation, progression, and resistance to therapy [17,18]. In clinical trials of patients with advanced CRC, low survival rates after chemotherapy have been closely linked to high expression levels of CSC markers [19,20]. The WNT/β-catenin pathway, activated within CSCs, plays a pivotal role in sustaining colorectal CSCs throughout CRC progression [21]. In addition, phosphorylated Akt and stabilized nuclear β-catenin initiate the transcription of CSC markers including c-Myc, CD44, and CD133, promoting cell survival and contributing to therapeutic resistance [16,22]. Consequently, the Wnt/β-catenin and Akt/mTOR signaling pathways are attractive targets for ameliorating drug resistance after chemotherapy [23].

Prenyl–phenol compounds isolated from the fungus *Ascochyta viciae* possess antiviral properties [24]. Among the prenyl–phenol antibiotics, 4-O-methyl-ascochlorin (MAC), a derivative of ascochlorin (ASC), is a potential anti-cancer agent due to its diverse physiological properties [25,26]. In particular, MAC triggers apoptosis by boosting caspase activity, inhibiting c-Myc in leukemia cells, and inducing autophagy via HIF-1α expression in lung cancer cells [26,27,28]. However, the effect of MAC on 5-FU-based therapy, drug resistance, and death of CRC cells is unclear.

In this study, we sought to determine whether MAC synergistically enhances the anti-tumor effects of 5-FU in the context of CRC. Furthermore, we examined whether MAC suppresses 5-FU resistance-induced anti-apoptosis and cancer stemness by inhibiting β-catenin, thereby overcoming CRC cell resistance to 5-FU.

## 2. Results

### 2.1. Synergistic Effects of Prenyl–Phenol Antibiotics and 5-FU on the Viability of CRC Cells

**Figure 1:**
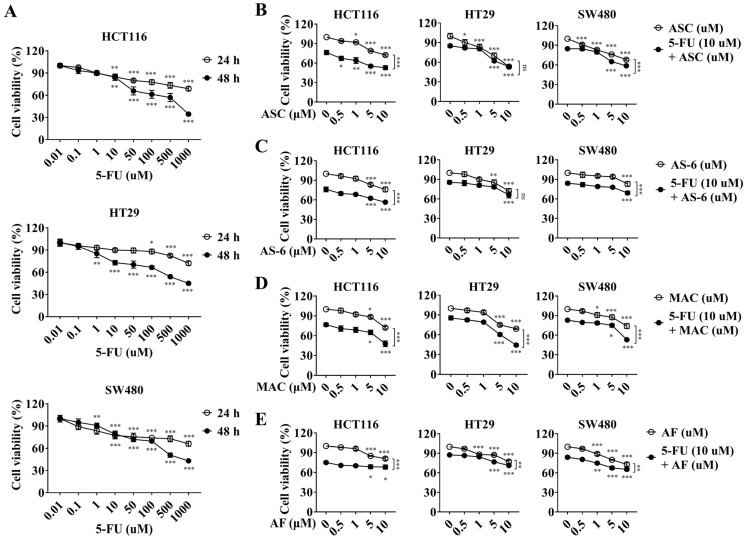
Synergistic effects of prenyl–phenol antibiotics and 5-FU on the viability of CRC cells. (**A**) HCT116, HT29, and SW480 cells were treated with indicated concentrations of 5FU for 24 and 48 h, respectively; then, the cells were exposed to an MTT solution for 3 h. (**B**–**E**) Cells were exposed to either 10 μM 5-FU, indicated concentrations of drugs ASC, AS-6, MAC, and AF alone or co-treated with 10 μM 5-FU for 48 h. All results were analyzed using two-way ANOVA. Mean ± SD, *n* = 3, *** *p* < 0.0001, ** *p* < 0.005, * *p* < 0.05 compared to non- or single treatment.

To evaluate 5-FU effects on HCT116, HT29, and SW480 CRC cells, we tested cell viability in an MTT assay. Treatment of CRC cells with 80 µM 5-FU for 24 h or 10 µM 5-FU for 48 h showed approximately 80% cell viability in all CRC lines (Figure 1A). For assays testing the combination of 5-FU and the novel compound, cells were exposed to 10 µM 5-FU for 48 h. To further evaluate the inhibitory and synergistic effects of prenyl–phenol antibiotics (ASC and AF) and ASC derivatives (AS-6 and MAC) on cell toxicity, CRC cells were treated with ASC, AS-6, MAC, or AF in the absence and presence of 5-FU. All drugs reduced cell viability in the absence of 5-FU. In HT-29 cells, 5-FU exhibited approximately 85% cell viability, whereas the combination treatment of ASC, AS-6, and AF with 5FU decreased cell viability similar to drugs used in monotherapy. However, treatment with MAC alone exhibited approximately 72% cell viability at a concentration of 10 μM, while co-treatment with MAC and 5-FU led to cell viability below 50% across all cell lines. These findings suggest that MAC shows synergistic effects when used with 5-FU in CRC (Figure 1B–E). Therefore, we further examined the molecular mechanisms underlying the synergistic effects of MAC and 5-FU on CRC cells’ proliferation and apoptosis.

### 2.2. MAC Enhances 5-FU-Induced Apoptosis of CRC Cells

**Figure 2:**
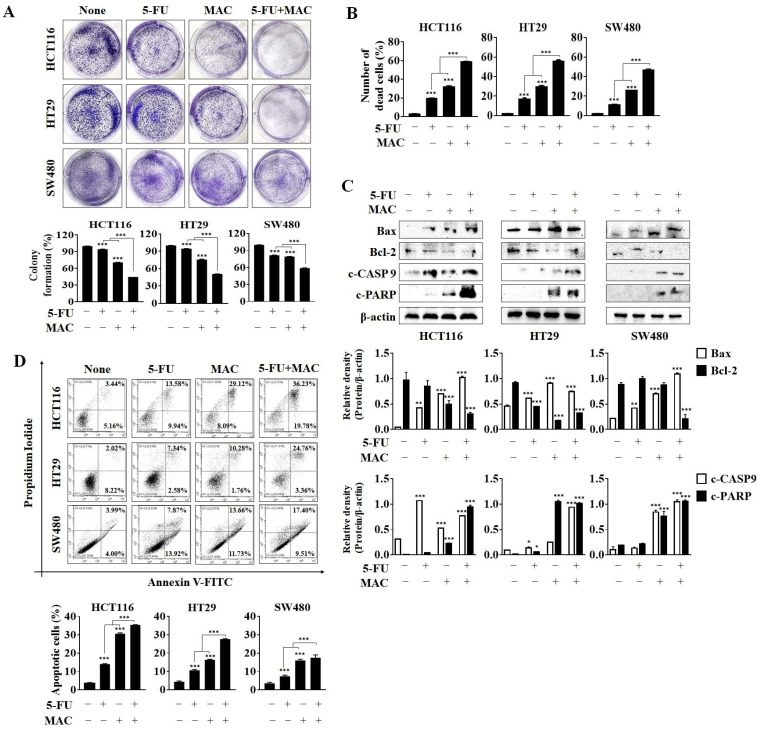
MAC enhances 5-FU-induced apoptosis in CRC cells. (**A**) Cell proliferation was determined by a colony formation assay after exposure to 5-FU (10 μM) and MAC (10 μM) alone or together for 48 h. (**B**) Cell death was confirmed by trypan blue staining after individual or concurrent treatment with 5-FU (10 μM) and MAC (10 μM) for 48 h. (**C**) Bax, Bcl-2, cleaved-caspase 9, and cleaved-PARP expression in CRC cells was detected by Western blot after an individual or concurrent treatment with 5-FU (10 μM) and MAC (10 μM). β-actin was employed as a loading control. (**D**) Death of CRC cells was detected by flow cytometry 48 h after individual or concurrent treatment with 5-FU (10 μM) and MAC (10 μM). All results were analyzed using one-way ANOVA. Mean ± SD, *n* = 3, * *p* < 0.05, ** *p* < 0.005, *** *p* < 0.0001 compared to a non- or single treatment.

The results of a colony formation assay revealed that co-treatment with MAC and 5-FU reduced CRC cells’ colony-forming ability to a much greater extent than MAC or 5-FU alone (Figure 2A). Trypan blue staining showed that MAC/5-FU increased cell death to a greater extent than treatment with either MAC or 5-FU alone (Figure 2B). Therefore, to ascertain whether MAC/5-FU-mediated cell death was apoptosis, we measured the expression of apoptosis markers. Co-treatment with MAC/5-FU led to a marked increase in the expression of cleaved caspase 9 and PARP compared to 5-FU alone (Figure 2C). Next, we measured the percentage of apoptotic cells by PI/annexin V staining. Similar to the results observed for apoptotic proteins, co-treatment with MAC/5-FU led to a marked increase in the percentage of apoptotic cells (Figure 2D). In HCT116 cells, 5-FU and MAC induced 23% and 37% of apoptotic cells, respectively, comprising both early and late apoptotic cells. Moreover, the combination treatment of 5-FU and MAC resulted in over 57% of apoptotic cells, demonstrating the highest synergistic effect among the three cell types. These results suggest that MAC enhances 5-FU-mediated apoptosis of CRC cells.

### 2.3. MAC Suppresses p53/21, Akt/mTOR/p70S6K, and Wnt/β-Catenin Signaling Pathways in CRC Cells

**Figure 3:**
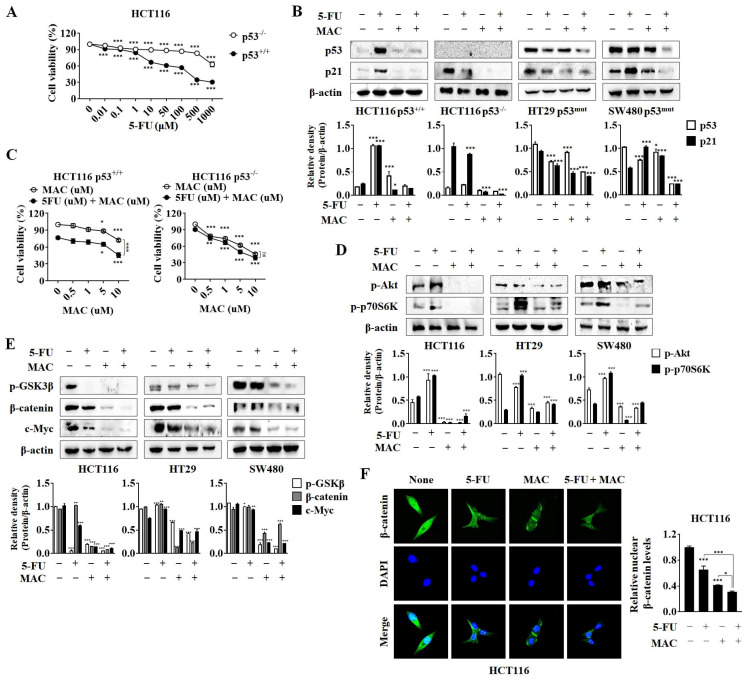
MAC suppresses the p53/21, Akt/mTOR/p70S6K and Wnt/β-catenin signaling pathways in CRC cells. (**A**) Viability of HCT116 p53^+/+^ and HCT116 p53^−/−^ cells was analyzed by MTT assay after treatment with 5-FU at indicated concentrations for 48 h. Results were analyzed using one-way ANOVA. (**B**) HCT116 p53^+/+^, HCT116 p53^−/−^, HT29 p53mut, and SW480 p53mut cells were exposed to 5-FU (10 μM) and MAC (10 μM) with each other or separately after 48 h. Western blot analysis was used to identify p53 and p21 protein levels, with β-actin serving as a loading control. (**C**) Viability of HCT116 p53^+/+^ and HCT116 p53^−/−^ cells was analyzed by MTT assay after administering indicated drug concentrations for 48 h. Results were analyzed using one-way ANOVA. (**D**,**E**) The protein levels of phosphor-Akt, -p70S6K, -GSK-3β, β-catenin, and c-Myc were assessed by Western blot analysis. (**F**) Nuclear β-catenin levels in HCT116 cells were detected by immunofluorescence after treatment with 5-FU (10 μM) or MAC (10 μM) for 48 h. The ImageJ program Version 1.54 was used to determine the levels of nuclear β-catenin. Results were analyzed using two-way ANOVA. Mean ± SD, *n* = 3, *** *p* < 0.0001, ** *p* < 0.005, * *p* < 0.05 compared to non- or single treatment.

5-FU is a well-known drug that induces apoptosis in CRC cells as a response to upregulation and accumulation of p53 [29]. As expected, the cytotoxic effects of 5-FU on HCT116 p53^−/−^ cells were less pronounced than those against HCT116 wild-type (WT) cells (Figure 3A). This finding suggests that the cytotoxic effects of 5-FU on CRC cells depend on p53 expression. Co-treatment with MAC and 5-FU clearly suppressed p53 and p21 expressions in HCT116 WT, HCT116 p53^−/−^, HT29 (p53^mut^), and SW480 (p53^mut^) cells (Figure 3B). However, the viability of HCT116 p53^−/−^ cells was reduced to a greater extent than HCT116 WT cells after MAC exposure (Figure 3C). These results suggest that MAC-induced inhibition of p53 expression is not related to MAC-induced inhibition of cell viability.

Next, we examined the effects of MAC and 5-FU on the Akt/mTOR/p70S6K and Wnt/β-catenin signaling pathways, which play critical roles in the growth and progression of CRC cells [30]. MAC decreased Akt and p70S6K phosphorylation significantly in the absence and presence of 5-FU (Figure 3D). In addition, MAC suppressed p-GSK3β, β-catenin, and c-Myc expression in all CRC cell lines (Figure 3E). Immunofluorescence analysis showed that β-catenin levels in the nucleus fell after treatment with either MAC or 5-FU (Figure 3F). Co-treatment with MAC and 5-FU suppressed the cytosolic and nuclear expression of β-catenin substantially. Overall, these findings suggest that MAC increases the anti-cancer activity of 5-FU by suppressing the phosphorylation of Akt/mTOR/p70S6K, as well as nuclear β-catenin expression in CRC cells.

### 2.4. MAC Reduces the Viability of 5-FU-Resistant (5-FU-R) HCT116 Cells

**Figure 4:**
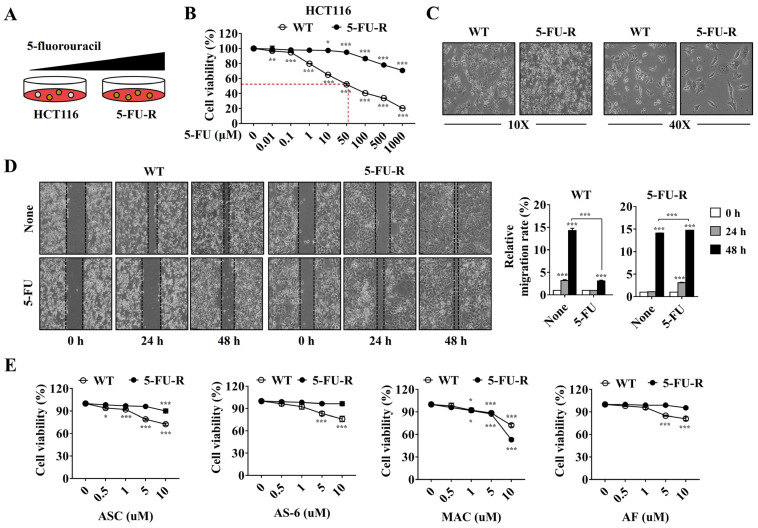
MAC inhibits cell viability in 5-FU-R cells compared to WT cells. (**A**) HCT116 cells were administered doses ranging from low to high to create 5-FU-R cells. (**B**) The viability of WT and 5-FU-R cells was analyzed by MTT after treatment with indicated concentrations of 5-FU for 48 h. (**C**) At magnifications of 10× and 40×, microscopic characteristics of the cell morphology were observed with photographs. (**D**) A wound healing assay was used to observe and compare the damage repair capacity of WT and 5-FU-R cells under 5-FU exposure conditions; ImageJ was used to quantify the movement distance. (**E**) 5-FU-R cells were treated with 0.5, 1, 5, and 10 μM of compounds (ASC, AS-6, MAC, and AF) alone or with 5-FU (10 μM) for 48 h. WT cells were used as a control. All results were analyzed using two-way ANOVA. Mean ± SD, *n* = 3, *** *p* < 0.0001, ** *p* < 0.005, * *p* < 0.05 compared to non- or single treatment.

5-FU-R cells were established by exposing HCT116 WT cells to low and high 5-FU concentrations for approximately 1 month (Figure 4A). At a concentration of 50 μM, which is the IC50 value in HCT116 WT, 5-FU reduced cell viability by only 5% in 5-FU-R. These results indicate that 5-FU-R cells have become resistant to 5-FU (Figure 4B). Recent studies report that drug-resistant cells exhibit a wide range of traits, including morphological changes and increased motility [31]. However, 5-FU-R cells did not exhibit distinguishable morphological variations from WT cells (Figure 4C). The migration rate of WT cells was lower after 5-FU treatment, whereas that of 5-FU-R cells was unaltered (Figure 4D). These findings suggest that 5-FU-R cells are stably resistant to 5-FU because 5-FU stimulation does not interfere with migration.

To overcome 5-FU resistance, we investigated the inhibitory effects of ASC, AS-6, MAC, and AF on 5-FU-R cell viability. ASC, AS-6, and AF had no effect on the viability of 5-FU-R cells, indicating that these drugs, similar to 5-FU, are resistant to 5-FU-R cells. By contrast, MAC exhibited approximately 72% cell viability in WT cells, whereas in 5FU-R cells, it exhibited approximately 53% cell viability (Figure 4E), indicating that MAC has more effective cell toxicity in 5FU-R cells than in WT cells. These findings suggest that MAC is a chemical compound with anti-cancer properties, and that it can be used to overcome CRC cells’ resistance to 5-FU.

### 2.5. MAC Triggers Apoptosis and Decreases Expression of CSC Markers in 5-FU-R Cells

**Figure 5:**
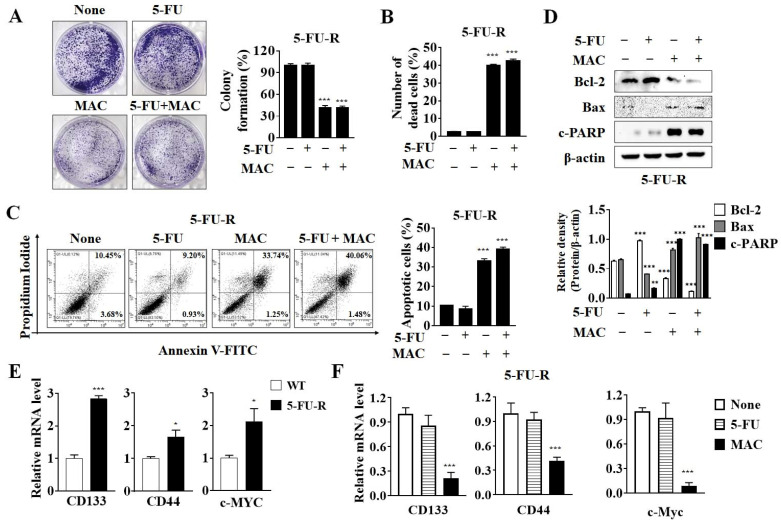
MAC triggers apoptosis and decreases CSC markers in 5-FU-R cells. (**A**) Cell proliferation was determined by a colony formation assay. (**B**) Cell death was determined by trypan blue staining. (**C**) Death of 5-FU-R cells was detected by cytometry after treatment with 5-FU (10 μM) or MAC (10 μM) for 48 h. (**D**) Western blot analysis was used to determine MAC’s (10 μM) alteration of the indicated proteins. (**E**) CD133, CD44, and c-Myc expression in WT and 5-FU-R cells were detected by real-time PCR. (**F**) After treatment with 5-FU (10 μM) or MAC (10 μM) for 48 h in 5-FU-R cells, CD133, CD44, and c-Myc expression were detected by real-time PCR. All results were analyzed using two-way ANOVA. Mean ± SD, *n* = 3, *** *p* < 0.0001, ** *p* < 0.005, * *p* < 0.05 compared to WT or non-treatment.

The inhibitory effects of MAC on the proliferation of 5-FU-R cells were confirmed in a colony formation assay. MAC suppressed colony formation in 5-FU-R cells (Figure 5A). Trypan blue staining revealed that MAC markedly increased the number of dead cells (Figure 5B), indicating that MAC inhibits cell proliferation and induces death in 5-FU-R cells. To confirm MAC-induced apoptosis in 5-FU-R cells, PI/Annexin V staining was performed, followed by flow cytometry analysis. Although 5-FU did not increase the number of apoptotic cells, MAC increased the number of both early and late apoptotic 5-FU-R cells in the absence or presence of 5-FU (Figure 5C). In addition, MAC increased Bax expression, cleaved PARP, and decreased bcl-2 expression (Figure 5D). These results strongly suggest that MAC promotes apoptotic cell death in 5-FU-R cells.

CSCs play a major role in resistance to chemotherapy and cancer relapse due to their self-renewal abilities. To determine whether 5-FU resistance is related to CSCs, we compared mRNA levels encoding CSC markers CD133, CD44, and c-Myc in WT and 5-FU-R cells. 5-FU-R cells expressed substantially higher levels of CSC markers than WT cells (Figure 5E), indicating that prolonged exposure to 5-FU may induce resistance among cells by enabling them to acquire CSC-like characteristics. Additionally, MAC markedly decreased the mRNA levels of CD133, CD44, and c-Myc in 5-FU-R cells (Figure 5F). These findings suggest that MAC overcomes 5-FU resistance by inducing apoptosis and suppressing CSC-like characteristics.

### 2.6. MAC Inhibits β-Catenin Activity in 5-FU-R Cells by Suppressing the PI3K/Akt/mTOR Signaling Pathways

**Figure 6:**
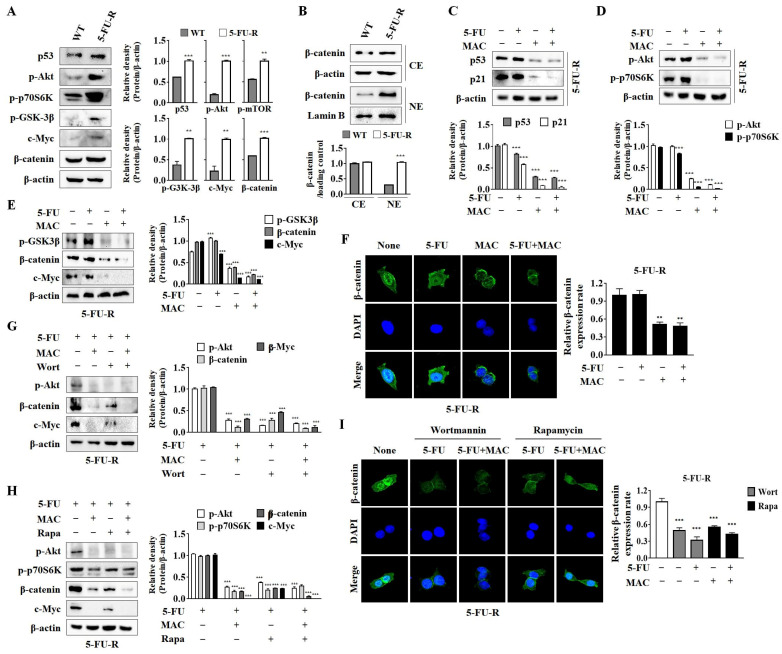
MAC inhibits β-catenin activity by suppressing PI3K/Akt/mTOR signaling pathways in 5-FU-R cells. (**A**,**B**) The protein levels of p53, phosphor-Akt, -p70S6K, -GSK3β, c-Myc, β-catenin, and both cytoplasmic and nucleic β-catenin in WT and 5-FU-R cells were obtained by Western blot analysis. (**C**–**E**) The protein levels of p53, p21, phosphor-Akt, -p70S6K, -GSK-3β, β-catenin, and c-Myc were obtained by Western blot analysis. (**F**,**I**) β-catenin (green) and DAPI (blue) were applied in an immunofluorescence investigation. β-catenin amounts were calculated using ImageJ. Mean ± SD, *n* = 3, *** *p* < 0.0001, ** *p* < 0.005 compared to non-treatment. Results were analyzed using two-way ANOVA. (**G**) In the presence of 5-FU (10 μM), 5-FU-R cells were stimulated with single or combination treatments of wortmannin (100 μM) or MAC (10 μM). (**H**) In the presence of 5-FU (10 μM), 5-FU-R cells were stimulated with single or combination treatments of rapamycin (0.1 μM) or MAC (10 μM). Western blot analysis was used to determine expression levels of phosphor-Akt, -p70S6K, β-catenin, and c-Myc.

Since MAC inhibits p53 expression as well as the Akt/mTOR and Wnt/β-catenin cascades in WT cells, we compared the expression levels of these oncogenic signaling proteins in WT and 5-FU-R cells. The protein levels of p53, p-Akt, p-p70S6K, p-GSK3β, c-Myc, and β-catenin were substantially higher in 5-FU-R cells than in WT cells (Figure 6A). In addition, 5-FU-R cells showed a clear elevation of β-catenin levels in the nucleus (Figure 6B). To further investigate the effects of MAC on p53, p-Akt, p-p70S6K, and β-catenin expression, we treated 5-FU-R cells with MAC and 5-FU. MAC reduced the activation of the p53/p21 pathway and Akt and p70S6K phosphorylation in 5-FU-R cells regardless of 5FU treatment (Figure 6C,D). MAC also inhibited β-catenin and c-Myc protein expression by activating GSK3β (Figure 6E). Immunofluorescence analyses revealed that the cytoplasmic and nuclear expression of β-catenin in 5-FU-R cells was suppressed by MAC (Figure 6F). Collectively, these results indicate that MAC inhibits the p53/p21, Akt/mTOR, and Wnt/β-catenin signaling pathways in 5-FU-R cells.

To confirm whether MAC inhibits β-catenin expression by suppressing the Akt/mTOR pathway, we treated 5-FU-R cells with wortmannin (Wort, a PI3K inhibitor) or rapamycin (Rapa, an mTOR inhibitor). Wort and Rapa inhibited β-catenin and c-Myc expression in 5FU-treated 5-FU-R cells (Figure 6G,H). Immunofluorescence analyses revealed that Wort and Rapa suppressed β-catenin levels in the cytoplasm and nucleus, similar to MAC (Figure 6I). Overall, MAC effectively reduced β-catenin activation in 5-FU-R cells by blocking the PI3K/Akt/mTOR signaling pathway.

### 2.7. MAC Induces Apoptosis and Inhibits Expression of CSC Marker in 5-FU-R Cells by Downregulating β-Catenin

**Figure 7:**
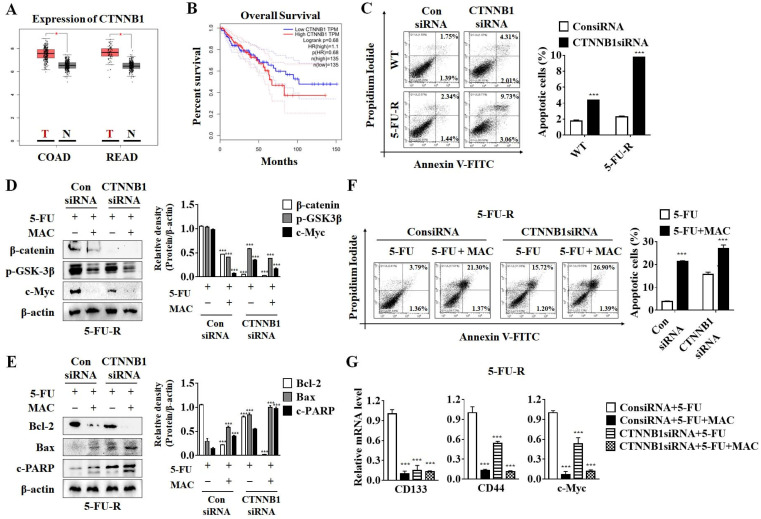
Downregulation of CTNNB1 expression is involved in MAC-mediated apoptosis and CSC markers inhibition in 5-FU-R cells. (**A**) Box plots of CTNNB1 mRNA expression in CRC from the GEPIA database. (**B**) The prognostic effect of CTNNB1 mRNA level in CRC from the GEPIA database. Dash lines represent the 95% confidence interval. (**C**) The number of apoptotic cells in 5-FU-R cells transfected with negative siRNA or CTNNB1 siRNA was measured by FACS analysis. (**D**,**E**) 5-FU-R cells transfected with negative or CTNNB1 siRNA were treated with 5-FU (10 μM) alone or co-treated with MAC (10 μM) and 5-FU (10 μM) for 48 h. Western blot analysis was used to determine the protein levels of β-catenin, phosphor-GSK3β, c-Myc, Bcl-2, Bax, and cleaved-PARP. (**F**) Negative control or CTNNB1 siRNA-transfected 5-FU-R cells were treated with MAC (10 μM) or 5-FU (10 μM) for 48 h. Then, the number of apoptotic cells was measured by FACS analysis after being stained with PI/Annexin-V. (**G**) The mRNA levels of CD133, CD44, and c-Myc were compared using real-time PCR. Values are averages of three separate studies’ standard deviations. All results were analyzed using two-way ANOVA. *** *p* < 0.0001, * *p* < 0.05 compared to the WT or control siRNA group.

According to a GEPIA data source, CTNNB1 expression, which encodes β-catenin, is higher in colon adenocarcinoma (COAD) and rectal adenocarcinoma cells than in normal cells (Figure 7A). CTNNB1 levels are associated with the overall survival of patients with COAD (Figure 7B). Therefore, we examined the involvement of β-catenin in WT and 5-FU-R cell survival using a siRNA targeting CTNNB1. CTNNB1 deficiency increased apoptotic cell death in both WT and 5-FU-R cells. Notably, CTNNB1 siRNA-transfected 5-FU-R cells showed a marked increase in apoptosis, indicating that 5-FU-R cells are more sensitive to β-catenin expression levels than WT cells (Figure 7C).

To determine whether suppressing the Wnt/β-catenin pathway is necessary to induce MAC-mediated apoptosis in 5-FU-R cells, we examined the GSK3β/β-catenin/c-Myc pathway and apoptotic factors in 5-FU-R cells transfected with CTNNB1 siRNA and then treated with MAC. In 5FuR cells, the suppression of β-catenin and c-Myc expression by CTNNB1 knockdown resembled that of cells treated with MAC. However, GSK3β was unaffected by CTNNB1 siRNA (Figure 7D). The expression levels of Bax and c-PARP increased in CTNNB1 knockdown cells, indicating that MAC-induced apoptosis is triggered by a decrease in β-catenin expression (Figure 7E). Furthermore, treatment with MAC in CTNNB1 knockdown cells increased the percentage of apoptotic cells more efficiently than either MAC or CTNNB1 siRNA alone (Figure 7F). These results suggest that β-catenin suppression plays a critical role in MAC-induced apoptosis of 5-FU-R cells. In addition, we investigated the association between MAC-reduced CSC markers (CD133, CD44, and c-Myc) and the Wnt/β-catenin pathway in CTNNB1 knockdown cells by real-time PCR. Deficiency of CTNNB1 in 5-FU-R cells suppressed CD133, CD44, and c-Myc mRNA levels. However, MAC inhibited CD44 and c-Myc mRNA expression in CTNNB1 knockdown cells (Figure 7G), suggesting that Wnt/β-catenin expression plays a role in reducing MAC-mediated stemness.

## 3. Discussion

Although 5-FU is still widely used as first-line chemotherapy for patients with CRC, the response rate is decreasing because nearly half of CRC tumors become resistant [5,32]. Resistance to chemotherapy remains a major challenge for patients with cancer. Many recent studies report that combination therapy is a good strategy for overcoming resistance to 5-FU therapy [33,34]. In this study, we evaluated the combined therapeutic effects of ASC, AF, and ASC derivatives (AS-6 and MAC) in addition to 5-FU on CRC cells in vitro. Among the drugs tested, MAC showed the strongest synergistic effects with 5-FU. Therefore, we explored the mechanisms underlying these anti-cancer effects.

5-FU-induced apoptosis is closely related to the expression of the tumor suppressor gene p53, whose overexpression makes CRC sensitive to 5-FU [35,36]. We found that 5-FU’s cytotoxic effects depended on increased p53 expression, and MAC abolished p53 expression in 5-FU-treated CRC cells, regardless of p53 mutations. p53 plays a dual role in cancer treatment, acting as a double-edged sword. Mutant p53, in particular, is an intriguing target for its promotion of tumor growth and contribution to therapy resistance [37,38]. Thus, we used HCT116 p53^−/−^ cells to investigate MAC’s role in response to p53 depletion. However, the synergistic effects of MAC and 5-FU on cell cytotoxicity were not observed in HCT116 p53^−/−^ cells. In summary, these results indicate that the cytotoxic effects of MAC in 5-FU-treated cells are not due to p53 suppression.

The PI3K/Akt/mTOR and Wnt/β-catenin signaling pathways are critical for CRC cells’ growth and proliferation [39,40]. Simultaneous activation of two critical pathways, Wnt signaling through the loss of the Wnt antagonist APC and constitutive PI3K activation, resulted in enhanced tumor initiation and promotion in mice [41]. Additionally, previous findings suggested that blocking the Wnt pathway may lead to compensatory activation of the Akt/mTOR pathway as a survival mechanism in CRC cells [40]. Thus, simultaneous modulation of both the Wnt and Akt/mTOR pathways may be a promising therapeutic strategy for CRC treatment. Our results showed that treatment with 5-FU increased p-Akt, p-mTOR, c-Myc, and β-catenin expression, whereas expression fell significantly in cells co-treated with MAC and 5-FU. These results suggest that MAC effectively attenuates both the Wnt and Akt/mTOR pathways, indicating its potential efficacy in inhibiting CRC.

The Wnt/β-catenin pathway not only enhances tumorigenicity but is also essential for drug resistance [12,42]. The Wnt inhibitor has been reported to significantly suppress 5-FU-R (HCT-8) cancer growth in mouse models [12]. CTNNB1 knockdown notably promotes apoptosis in 5-FU-R cells, which is consistent with previous reports where Wnt inhibitors restored cell sensitivity to 5-FU. Furthermore, MAC suppresses the Wnt/β-catenin pathway by inhibiting the Akt/mTOR pathway in 5-FU-R cells. These findings indicate that β-catenin inhibition is a major target for triggering MAC-induced apoptosis in cells resistant to 5-FU.

CSCs contribute to drug resistance in CRC, leading to both cancer survival and recurrence [43,44]. Wnt/β-catenin signaling plays an important role in the initial activation and self-renewal of CSCs [45]. A recent study demonstrated that p53 activates the WNT/β-catenin signaling pathway in cells exposed to 5-FU, leading to CSC activation within tumors [6]. 5-FU-R cells exhibited higher p53 expression, Wnt/β-catenin signaling pathways, and CSC markers including CD44, CD133, and c-Myc than WT cells. MAC suppressed the expression of CSC markers, p53, and β-catenin in 5-FU-R cells. These results suggest that MAC-induced inhibition of the Wnt/β-catenin pathway abolishes 5-FU resistance mediated by CSCs. Although MAC-induced inhibition of p53 did not affect 5-FU-regulated cell viability, inhibition may be related to the reduced expression of CSC markers in 5-FU-R cells.

**Figure 8:**
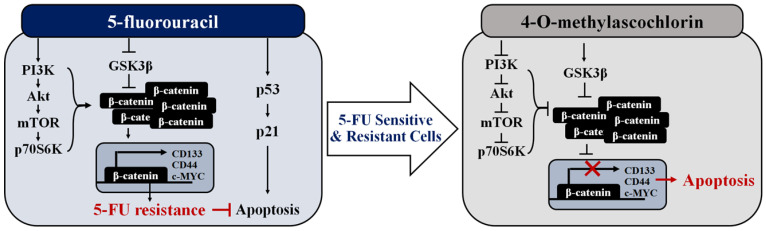
Schematic model for MAC’s anti-tumor activity with 5-FU in 5-FU-sensitive and 5-FU-resistant CRC cells.

In summary, the data presented herein show that MAC has great potential as a therapeutic adjuvant to 5-FU for treating CRC cells. MAC enhanced 5-FU-induced apoptosis by inhibiting β-catenin expression via reduced Akt/mTOR signaling (Figure 8). Importantly, MAC exerted various anti-tumor effects that reduced 5-FU-R cells’ stemness in addition to its ability to induce apoptosis. These data suggest that MAC is an efficient adjuvant candidate for 5-FU-based therapy in CRC cells.

## 4. Materials and Methods

### 4.1. Cells and Materials

HCT116, HT29, and SW480 CRC cells were obtained from the American Type Culture Collection (Manassas, VA, USA). HCT116, HT29, and SW480 were cultured in DMEM and RPMI (HyClone; GE Healthcare Life Sciences, Logan, UT, USA), including 10% FBS (HyClone) and 1% antibiotic. 5-FU-R cells were established by exposing HCT116 cells to doses of 0.01–80 μM 5-FU for roughly 2 months to stabilize resistance. After the cells were obtained, they were validated and maintained for less than 20 passages and used for experiments. The formulations for prenyl–phenol antibiotics (ASC and ascofuranene (AF)) and ASC derivatives (4-O-carboxymethylascochlorin (AS-6) and MAC) were provided by Chugai Pharmaceutical (Tokyo, Japan). 5-FU was purchased from Sigma-Aldrich Company (Saint Louis, MO, USA).

### 4.2. Cell Viability Assay

CRC cells were seeded at a density of 2 × 10^4^ cells/well in 96-well plates and incubated overnight. CRC cells were incubated with a medium containing 5-FU at concentrations of 0, 0.01, 0.1, 1, 10, 50, 100, 500, and 1000 μM in each well for 24 and 48 h. Cells were exposed to indicated drugs (ASC, AS-6, MAC, and AF) alone and in combination with 10 μM 5-FU for 48 h. Then, the formazan crystals were solubilized by adding DMSO following treatment with an MTT (5-diphenyl-2H-tetrazolium bromide) working solution for 3 h. At 540 nm, absorbance was measured by a microplate reader (VersaMaxTM Microplate Reader Molecular Devices, LLC, Sunnyvale, CA, USA).

### 4.3. Colony Formation Assay

CRC cells were seeded in 6-well plates (2 × 10^3^ cells/well) and incubated until they reached 60% confluence. Cells were treated with 10 μM 5-FU, 10 μM MAC, or both treatments for 48 h. Then, they were fixed with 100% methanol for 3 min and stained with 0.5% crystal violet for 30 min for photography.

### 4.4. Trypan Blue Staining

Cells were seeded in a 60 mm dish (5 × 10^5^ cells/well) and incubated overnight. Cells were treated with 10 μM 5-FU, 10 μM MAC, or both treatments for 48 h. The harvested cells were combined for 5 min at room temperature with a 0.4% trypan blue solution (Sigma-Aldrich). In the mixture of trypan blue-cell suspension in the hemocytometer chamber, stained and non-stained cells were counted using a microscope (Nikon Eclipse TS100, EquipNet, Inc., Canton, MA, USA).

### 4.5. Flow Cytometry Analysis

Cells (5 × 10^5^ cells) were seeded in 60 mm dishes and treated with 10 µM of 5-FU and 10 µM of MAC for 48 h. According to the manufacturer’s instructions, all cells were collected using trypsin-EDTA, stained with a FITC annexin V kit from BD Pharmingen in NJ, and then analyzed using flow cytometry (Beckman Coulter, Miami, FL, USA).

### 4.6. Western Blot Analysis

Cells (5 × 10^5^ cells) were seeded in 60 mm dishes and incubated overnight. Cells were cultured with the indicated drugs for 48 h. Harvested cells were lysed in a lysis buffer. Protein was loaded onto SDS-PAGE and transferred to an Immobilon-P-membrane (Millipore, Burlington, MA, USA). After blocking the membrane with 5% non-fat milk for 30 min, it was incubated overnight at 4 °C with w-7480; Santa Cruz Biotechnology, Inc., Dallas, TX, USA), Bcl-2 (sc-7382), p53 (sc-126), P21 (sc-397), p-GSK-3β (sc-11757), β-catenin (sc-7963), c-Myc (sc-764), phosphorylated(p)-AKT (sc-7383), p-P70S6K (sc-7984), β-actin (sc-47778), cleaved(c)-PARP (#5625; Cell signaling Technology, Inc., Danvers, MA, USA), c-caspase 8 (#8592), c-caspase 9 (#7237), c-caspase 3 (#9661), and c-caspase 7 (#9491). The membranes were exposed for 2 h at RT with secondary antibodies. After being washed three times with TBS-T, chemiluminescence (GE Healthcare, Chicago, IL, USA) was used to visualize the reaction and identified by ChemiDOCTM XRS (Bio-Rad Laboratories, Hercules, CA, USA).

### 4.7. Immunofluorescence Assay

HCT116 and 5-FU-R cells of 5 × 10^4^ were cultured on 8 chamber slides and incubated overnight. HCT116 and 5-FU-R cells were treated with indicated drug concentrations for 48 h. Cells were fixed with 100% methanol for 1 min, permeabilized with 0.2% Triton X-100 for 5 min, and blocked with 10% normal goat serum (Thermo Fisher Scientific, Waltham, CA, USA). Cells were incubated with primary antibodies against β-catenin (Santa Cruz Biotechnology), reacted with Alexa Fluor 488-conjugated anti-mouse IgG and Flamma 648-conjugated anti-rabbit secondary antibodies (Thermo Fisher Scientific), and stained with DAPI (Sigma-Aldrich, St. Louis, MO, USA). Fluorescent impulses were visualized with a confocal microscope (Leica Microsystems GmbH, Wetzlar, Germany).

### 4.8. Wound Healing Assay

Cells were seeded in a 60 mm culture dish (5 × 10^5^ cells/dish) and incubated overnight. A 200 µL pipette tip was used to scrape an artificial vertical line. Cells were incubated for 48 h with a medium containing 10 μM of -FU. Images of the wound closure were captured using a Nikon Eclipse TS100 microscope.

### 4.9. Real-Time Polymerase Chain Reaction

In a 60 mm plate, 5 × 10^5^ HCT116 and 5-FU-R cells were seeded. Cells were administered the relevant concentrations of 5-FU, MAC, or CTNNB1 siRNA. According to the procedure, RNA was extracted using the TRIzol reagent from Thermo Fisher Scientific, Inc. After cDNA synthesis, SYBR green PCR master mix (Cosmo Genetech, Daejeon, Republic of Korea) and c-Myc: 5′-CCT GGT GCT CCA TGA GGA GAC-3′, 5′-CAG ACT CTG ACC TTT TGC CAG G-3′, CD44: 5′-CCA GAA GGA ACA GTG GTT RGG C-3′, 5′-ACT GTC CTC TGG GCT TGG TGT T-3′, and CD133: 5′-CAC TAC CAA GGA CAA GGC GTT C-3′, 5′-CAA CGC CTC TTT GGT CTC CTT G-3′ primers (Bioneer OCO., Daejeon, Republic of Korea) were performed with Real-Time PCR instrument (Bio-Rad Laboratories Inc., Hercules, CA, USA).

### 4.10. Gene Knockdown

Following the procedure, G-fectin (Gene solution, Kyungki-do, Republic of Korea) was used to transfect 5-FU-R cells with CTNNB1 siRNA and a negative control siRNA. The specific sequence was as follows: sense, 5′-GUUAUGGUCCAUCAGCUUUdTdT-3′, and antisense, 5′-AAAGCUGAUGGACCAUAACdTdT-3′. After 24 h, the cells were incubated with 5-FU and MAC for 48 h.

### 4.11. Statistical Analysis

All in vitro research employed data from at least three different, triplicate-run experiments. Statistical significance was determined using one-way or two-way ANOVA in Graph Pad Prism 7 (Graph Pad Software, Inc., La Jolla, CA, USA). Data were indicated as mean ± standard error. Statistically significant differences between the experimental and control or single treatment values were set at *** *p* < 0.0001, ** *p* < 0.005, and * *p* < 0.05.

## Data Availability

Data is contained within the article.

## References

[1] Siegel R.L., Miller K.D., Jemal A. (2019). Cancer statistics, 2019. CA Cancer J. Clin..

[2] Pita-Fernandez S., Alhayek-Ai M., Gonzalez-Martin C., Lopez-Calvino B., Seoane-Pillado T., Pertega-Diaz S. (2015). Intensive follow-up strategies improve outcomes in nonmetastatic colorectal cancer patients after curative surgery: A systematic review and meta-analysis. Ann. Oncol..

[3] Hernandez Dominguez O., Yilmaz S., Steele S.R. (2023). Stage IV Colorectal Cancer Management and Treatment. J. Clin. Med..

[4] Sethy C., Kundu C.N. (2021). 5-Fluorouracil (5-FU) resistance and the new strategy to enhance the sensitivity against cancer: Implication of DNA repair inhibition. Biomed. Pharmacother..

[5] Vodenkova S., Buchler T., Cervena K., Veskrnova V., Vodicka P., Vymetalkova V. (2020). 5-fluorouracil and other fluoropyrimidines in colorectal cancer: Past, present and future. Pharmacol. Ther..

[6] Cho Y.-H., Ro E.J., Yoon J.-S., Mizutani T., Kang D.-W., Park J.-C., Il Kim T., Clevers H., Choi K.-Y. (2020). 5-FU promotes stemness of colorectal cancer via p53-mediated WNT/β-catenin pathway activation. Nat. Commun..

[7] Chen X., Wang Y., Xia H., Wang Q., Jiang X., Lin Z., Ma Y., Yang Y., Hu M. (2012). Loss of E-cadherin promotes the growth, invasion and drug resistance of colorectal cancer cells and is associated with liver metastasis. Mol. Biol. Rep..

[8] Azwar S., Seow H.F., Abdullah M., Faisal Jabar M., Mohtarrudin N. (2021). Recent Updates on Mechanisms of Resistance to 5-Fluorouracil and Reversal Strategies in Colon Cancer Treatment. Biology.

[9] Clevers H., Nusse R. (2012). Wnt/beta-catenin signaling and disease. Cell.

[10] Jarde T., Evans R.J., McQuillan K.L., Parry L., Feng G.J., Alvares B., Clarke A.R., Dale T.C. (2013). In vivo and in vitro models for the therapeutic targeting of Wnt signaling using a Tet-ODeltaN89beta-catenin system. Oncogene.

[11] Schatoff E.M., Leach B.I., Dow L.E. (2017). Wnt Signaling and Colorectal Cancer. Curr. Color. Cancer Rep..

[12] He L., Zhu H., Zhou S., Wu T., Wu H., Yang H., Mao H., SekharKathera C., Janardhan A., Edick A.M. (2018). Wnt pathway is involved in 5-FU drug resistance of colorectal cancer cells. Exp. Mol. Med..

[13] Johnson S.M., Gulhati P., Rampy B.A., Han Y., Rychahou P.G., Doan H.Q., Weiss H.L., Evers B.M. (2010). Novel expression patterns of PI3K/Akt/mTOR signaling pathway components in colorectal cancer. J. Am. Coll. Surg..

[14] Xia P., Xu X.-Y. (2015). PI3K/Akt/mTOR signaling pathway in cancer stem cells: From basic research to clinical application. Am. J. Cancer Res..

[15] Patergnani S., Buchsbaum D., Piazza G. (2022). Targeting the Wnt/β-catenin signaling pathway in cancer. Front. Oncol..

[16] Yang L., Shi P., Zhao G., Xu J., Peng W., Zhang J., Zhang G., Wang X., Dong Z., Chen F. (2020). Targeting cancer stem cell pathways for cancer therapy. Signal Transduct. Target Ther..

[17] Batlle E., Clevers H. (2017). Cancer stem cells revisited. Nat. Med..

[18] Carnero A., Garcia-Mayea Y., Mir C., Lorente J., Rubio I.T., ME L.L. (2016). The cancer stem-cell signaling network and resistance to therapy. Cancer Treat. Rev..

[19] Eppert K., Takenaka K., Lechman E.R., Waldron L., Nilsson B., Van Galen P., Metzeler K.H., Poeppl A., Ling V., Beyene J. (2011). Stem cell gene expression programs influence clinical outcome in human leukemia. Nat. Med..

[20] Giuliano M., Giordano A., Jackson S., Hess K.R., De Giorgi U., Mego M., Handy B.C., Ueno N.T., Alvarez R.H., De Laurentiis M. (2011). Circulating tumor cells as prognostic and predictive markers in metastatic breast cancer patients receiving first-line systemic treatment. Breast Cancer Res..

[21] Moon B.S., Jeong W.J., Park J., Kim T.I., Min do S., Choi K.Y. (2014). Role of oncogenic K-Ras in cancer stem cell activation by aberrant Wnt/β-catenin signaling. J. Natl. Cancer Inst..

[22] Brossa A., Papadimitriou E., Collino F., Incarnato D., Oliviero S., Camussi G., Bussolati B. (2018). Role of CD133 Molecule in Wnt Response and Renal Repair. Stem Cells Transl. Med..

[23] Cheng X., Xu X., Chen D., Zhao F., Wang W. (2019). Therapeutic potential of targeting the Wnt/beta-catenin signaling pathway in colorectal cancer. Biomed. Pharmacother..

[24] Tamura G., Suzuki S., Takatsuki A., Ando K., Arima K. (1968). Ascochlorin, a new antibiotic, found by the paper-disc agar-diffusion method. I. Isolation, biological and chemical properties of ascochlorin. (Studies on antiviral and antitumor antibiotics. I). J. Antibiot..

[25] Tsuruga M., Nakajima H., Ozawa S., Togashi M., Chang Y.-C., Ando K., Magae J. (2004). Characterization of 4-O-methyl-ascochlorin-induced apoptosis in comparison with typical apoptotic inducers in human leukemia cell lines. Apoptosis.

[26] Jeong J.-H., Kang J.H., Hwang S.-L., Cho H.-J., Park K.-K., Park Y.-Y., Chung I.-K., Chang H.-W., Kim C.-H., Min K.-S. (2011). 4-O-methylascochlorin, methylated derivative of ascochlorin, stabilizes HIF-1α via AMPK activation. Biochem. Biophys. Res. Commun..

[27] Shin J.M., Jeong Y.J., Cho H.J., Magae J., Bae Y.S., Chang Y.C. (2016). Suppression of c-Myc induces apoptosis via an AMPK/mTOR-dependent pathway by 4-O-methyl-ascochlorin in leukemia cells. Apoptosis.

[28] Seok J.Y., Jeong Y.J., Hwang S.K., Kim C.H., Magae J., Chang Y.C. (2018). Upregulation of AMPK by 4-O-methylascochlorin promotes autophagy via the HIF-1alpha expression. J. Cell Mol. Med..

[29] Yang Y., Zhang M., Zhang Y., Liu K., Lu C. (2023). 5-Fluorouracil Suppresses Colon Tumor through Activating the p53-Fas Pathway to Sensitize Myeloid-Derived Suppressor Cells to FasL(+) Cytotoxic T Lymphocyte Cytotoxicity. Cancers.

[30] Ghafouri-Fard S., Abak A., Tondro Anamag F., Shoorei H., Fattahi F., Javadinia S.A., Basiri A., Taheri M. (2021). 5-Fluorouracil: A Narrative Review on the Role of Regulatory Mechanisms in Driving Resistance to This Chemotherapeutic Agent. Front. Oncol..

[31] Dratkiewicz E., Simiczyjew A., Pietraszek-Gremplewicz K., Mazurkiewicz J., Nowak D. (2019). Characterization of Melanoma Cell Lines Resistant to Vemurafenib and Evaluation of Their Responsiveness to EGFR- and MET-Inhibitor Treatment. Int. J. Mol. Sci..

[32] Mhaidat N.M., Bouklihacene M., Thorne R.F. (2014). 5-Fluorouracil-induced apoptosis in colorectal cancer cells is caspase-9-dependent and mediated by activation of protein kinase C-delta. Oncol. Lett..

[33] Tong J., Xie G., He J., Li J., Pan F., Liang H. (2011). Synergistic antitumor effect of dichloroacetate in combination with 5-fluorouracil in colorectal cancer. J. Biomed. Biotechnol..

[34] Alnuqaydan A.M., Rah B., Almutary A.G., Chauhan S.S. (2020). Synergistic antitumor effect of 5-fluorouracil and withaferin-A induces endoplasmic reticulum stress-mediated autophagy and apoptosis in colorectal cancer cells. Am. J. Cancer Res..

[35] Pereira D.M., Simões A.E., Gomes S.E., Castro R.E., Carvalho T., Rodrigues C.M., Borralho P.M. (2016). MEK5/ERK5 signaling inhibition increases colon cancer cell sensitivity to 5-fluorouracil through a p53-dependent mechanism. Oncotarget.

[36] Yang C.M., Kang M.-K., Jung W.-J., Joo J.-S., Kim Y.-J., Choi Y., Kim H.-P. (2021). p53 expression confers sensitivity to 5-fluorouracil via distinct chromatin accessibility dynamics in human colorectal cancer. Oncol. Lett..

[37] Murray-Zmijewski F., Slee E.A., Lu X. (2008). A complex barcode underlies the heterogeneous response of p53 to stress. Nat. Rev. Mol Cell Biol.

[38] Muller P.A., Vousden K.H. (2013). p53 mutations in cancer. Nat. Cell Biol..

[39] Bienz M., Clevers H. (2000). Linking colorectal cancer to Wnt signaling. Cell.

[40] Prossomariti A., Piazzi G., Alquati C., Ricciardiello L. (2020). Are Wnt/beta-Catenin and PI3K/AKT/mTORC1 Distinct Pathways in Colorectal Cancer?. Cell. Mol. Gastro. Hep..

[41] Deming D.A., Leystra A.A., Nettekoven L., Sievers C., Miller D., Middlebrooks M., Clipson L., Albrecht D., Bacher J., Washington M.K. (2014). PIK3CA and APC mutations are synergistic in the development of intestinal cancers. Oncogene.

[42] Katoh M., Katoh M. (2022). WNT signaling and cancer stemness. Essays Biochem..

[43] Borovski T., De Sousa E.M.F., Vermeulen L., Medema J.P. (2011). Cancer stem cell niche: The place to be. Cancer Res..

[44] Wang M.-Y., Qiu Y.-H., Cai M.-L., Zhang C.-H., Wang X.-W., Liu H., Chen Y., Zhao W.-L., Liu J.-B., Shao R.-G. (2020). Role and molecular mechanism of stem cells in colorectal cancer initiation. J. Drug Target.

[45] Zhu G.-X., Gao D., Shao Z.-Z., Chen L., Ding W.-J., Yu Q.-F. (2021). Wnt/β-catenin signaling: Causes and treatment targets of drug resistance in colorectal cancer. Mol. Med. Rep..

